# Presynaptic partner selection during retinal circuit reassembly varies with timing of neuronal regeneration *in vivo*

**DOI:** 10.1038/ncomms10590

**Published:** 2016-02-03

**Authors:** Takeshi Yoshimatsu, Florence D. D'Orazi, Clare R. Gamlin, Sachihiro C. Suzuki, Arminda Suli, David Kimelman, David W. Raible, Rachel O. Wong

**Affiliations:** 1 Department of Biological Structure, University of Washington, 1959 NE Pacific Street, Box 357420, Seattle, Washington 98195, USA; 2 Department of Physiology and Developmental Biology, Brigham Young University, 3048 LSB, Provo, Utah 84602, USA; 3 Department of Biochemistry, University of Washington, 1705 NE Pacific Street, Box 357350, Seattle, Washington 98195, USA

## Abstract

Whether neurons can restore their original connectivity patterns during circuit repair is unclear. Taking advantage of the regenerative capacity of zebrafish retina, we show here the remarkable specificity by which surviving neurons reassemble their connectivity upon regeneration of their major input. H3 horizontal cells (HCs) normally avoid red and green cones, and prefer ultraviolet over blue cones. Upon ablation of the major (ultraviolet) input, H3 HCs do not immediately increase connectivity with other cone types. Instead, H3 dendrites retract and re-extend to contact new ultraviolet cones. But, if regeneration is delayed or absent, blue-cone synaptogenesis increases and ectopic synapses are made with red and green cones. Thus, cues directing synapse specificity can be maintained following input loss, but only within a limited time period. Further, we postulate that signals from the major input that shape the H3 HC's wiring pattern during development persist to restrict miswiring after damage.

## Abstract (web summary)

Neurons in the zebrafish retina regenerate. Here, Yoshimatsu and colleagues show that retinal horizontal cells maintain their synaptic preferences for a limited period before circuit remodeling is triggered after photoreceptor loss.

## Graphical abstract

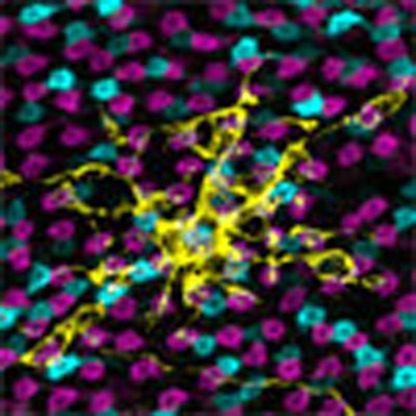

Circuits across the nervous system are highly complex, comprising stereotypic numbers of convergent and divergent connections between multiple, but specific pre- and postsynaptic cell types. Studies across diverse model organisms have uncovered many of the cellular and molecular mechanisms that shape such specificity in connectivity during development^1^,^2^,^3^. In contrast, our understanding of how well neuronal circuits are able to recreate their unique connectivity patterns after disease or injury remains scarce. In particular, it is unclear whether neurons that survive in a perturbed network maintain specificity in their synaptic partner choices, and are able to recapture their original wiring pattern upon circuit reassembly.

Here we took advantage of the intrinsic regenerative capacity of zebrafish to track the reassembly of neuronal circuits *in vivo*. Retinal horizontal cells (HCs) in general regulate glutamate transmitter release from photoreceptors^4^,^5^,^6^. We focused on a single type of HC, the H3 cell, in the zebrafish retina that makes a stereotypic pattern of connections with cone photoreceptors, synapsing with ultraviolet and blue, but not red or green, cones^7^,^8^. Moreover, H3 HCs favour ultraviolet over blue cones, contacting about five ultraviolet cones to one blue cone by 10 days post fertilization (d.p.f.), several days after the onset of visual behaviour^8^. Cone-type selectivity is attained by elimination of early red- and green-cone contacts, followed by preferential synaptogenesis with ultraviolet cones compared with blue cones, a process requiring ultraviolet-cone transmission. Therefore, in this circuit, synapse elimination initially refines synaptic partner choice, and selective synapse formation subsequently establishes the desired input convergence with the appropriate presynaptic partner types. This combination of cellular strategies engaged in the assembly of H3 HC–cone circuits led us to ask: (i) do H3 HCs retain specificity in their cone partner choice, maintaining a preference for ultraviolet cones over blue cones, and avoiding red and green cones during circuit repair?; (ii) is the developmental sequence of selective synapse formation and elimination that generates the synaptic convergence pattern of the H3 HCs re-engaged during regeneration? To answer these questions, we selectively ablated ultraviolet or blue cones after the initial assembly of HC–cone photoreceptor circuits, and waited for cone regeneration and the H3 HC circuit to reform *in vivo*.

We generated transgenic fish in which ultraviolet or blue cones were labelled and could be ablated selectively and with temporal control. Ablation triggers transdifferentiation of resident Müller glial cells to produce new neurons within a few days^9^. We examined the connectivity of H3 HCs with newly generated and previously existing cones. Multiphoton time-lapse imaging was carried out to monitor dynamic changes in the H3 HC dendrites during ultraviolet-cone loss and during reconstruction of the circuit. In addition, we compared H3 HC connectivity patterns across three conditions: when ultraviolet cones are regenerated rapidly, when regeneration is delayed and when regeneration is prevented. Findings from these comparisons led us to conclude that surviving neurons can maintain specificity in their preference of synaptic partners, but only within a limited time window.

## Results

### Ultraviolet-cone ablation triggers cone regeneration

**Figure 1:**
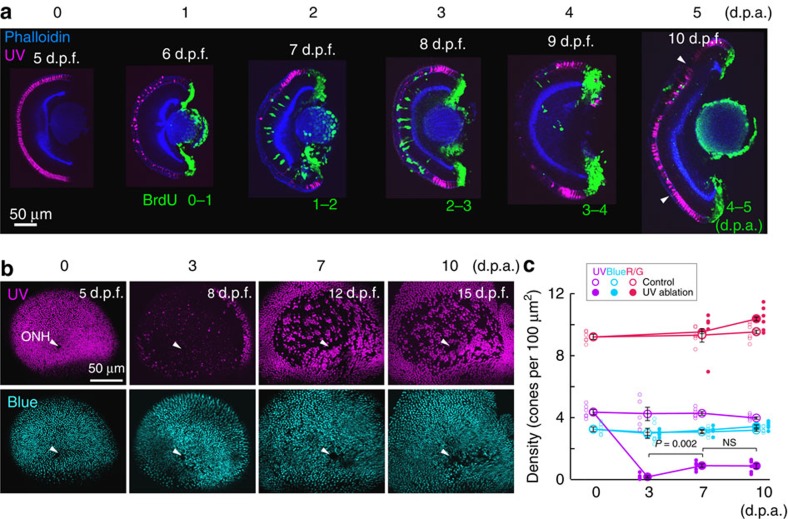
Ultraviolet-cone ablation triggers cone regeneration. (**a**) Hemisected retina before (at 5 days post fertilization, d.p.f.) and after 1–5 days post ablation (p.d.a.). Ultraviolet cones (magenta) were visualized in *Tg(sws1:nfsB-mCherry)*. BrdU (green) was applied for 1 day at various time points after ablation. The hemisected eyes were stained for phalloidin (blue) to mark in the inner and outer synaptic layers. Arrowheads indicate boundary between original ablated area and the region where ultraviolet cones were added subsequently due to continual retinal growth. (**b**) Whole-mount retina from *Tg(sws1:nfsB-mCherry, sws2:GFP)* fish fixed at indicated time points, showing *en face* view of ultraviolet (magenta) and blue (cyan) cones before and after ultraviolet-cone ablation and regeneration. Arrowheads point to the location of the optic nerve head (ONH). (**c**) Cone densities before and after ultraviolet-cone ablation, and from age-matched control animals. Large circles are the mean values and small circles indicate values from each retina. Error bars are s.e.m. Red and green cones (R/G) were visualized by immunostaining with the zpr1 antibody. NS, not significant (*P*=0.94). *P* values are from Mann–Whitney rank-sum test. UV, ultraviolet.

We selectively targeted ultraviolet cones in the zebrafish retina for ablation by creating a stable transgenic line in which the bacterial nitroreductase enzyme (NTR; *nfsb*) is expressed under the control of the *Opn1sw1* promoter^10^. This enzyme converts the prodrug metronidazole (Met) into a cytotoxin, killing cells that express NTR^11^,^12^. NTR was tagged with the fluorescent protein (FP), mCherry, enabling visualization of cells expressing the transgene. After treating zebrafish at 5 d.p.f. with Met for 2 h, most ultraviolet cones were ablated, and cell debris was cleared within 3 days post ablation (d.p.a.; Fig. 1a,b). We found that ultraviolet-cone ablation was specific because the densities of blue (*Tg(sws2:GFP)*) and zpr1 immunopositive red and green cones were not significantly different from those of controls (Fig. 1c).

Ultraviolet cones repopulated the region of ablation over time (Fig. 1a,b). This recovery plateaued by 7 d.p.a., reaching a value of ∼25% of the original ultraviolet population (Fig. 1c). Exposure of Met-treated animals to bromodeoxyuridine (BrdU) for 24 h before fixation at various time points between 0 and 5 d.p.a. revealed that cell genesis in the ablation region largely occurred 1–3 days after ultraviolet-cone death (Fig. 1a). This observation is consistent with previous studies showing that robust cell genesis occurs in zebrafish within 4 days after large-scale retinal damage^13^. Differentiated ultraviolet cones clearly occupied the outer nuclear layer by 5 d.p.a. (Fig. 1a). To confirm that these cells were indeed generated after the period of cell loss, we performed (2′S)-2′-deoxy-2′-fluoro-5-ethynyluridine (EdU) labelling between 1 and 4 d.p.a., and determined the identity of EdU-positive-cone photoreceptors. We found that within the central retina, there were no EdU-positive ultraviolet cones in non-Met-treated animals (control), whereas almost half the ultraviolet cones were labelled by EdU in Met-treated animals (Supplementary Fig. 1a,b). Ultraviolet cones that were not EdU positive may have survived the Met treatment or may have been generated outside the window of EdU application. Of the EdU-labelled nuclei in the photoreceptor layer, about half were zpr1-positive red/green cones, but very few were blue cones (Supplementary Fig. 1c,d). Because the numbers of regenerated red/green or blue cones were small compared with their original populations, there was no significant change in the densities of these cone types in the zone of regeneration (Fig. 1c; Supplementary Fig. 1). Together, our quantitative analysis indicates that selective ablation of ultraviolet cones triggers ultraviolet-cone genesis, and although cone regeneration was not exclusive to this cone type, production of other cone types during regeneration did not significantly alter their respective distributions.

### Blue-cone contact is unaltered as HCs rewire

We next determined whether H3 HCs were able to connect with the regenerated population of ultraviolet cones. Isolated H3 HCs were labelled by FP expression driven by the *Cx55.5* promoter^8^. To unequivocally identify regenerated cones, the animals were immersed in EdU containing fish media between 6 and 9 d.p.f. (1–4 d.p.a.). Confocal reconstructions of the H3 HCs and the surrounding cone photoreceptors were obtained after ultraviolet cones repopulated the retina at 15 d.p.f. (10 d.p.a.). As in a previous study^8^, synaptic contacts between HCs and cones were defined by dendritic invaginations into the cone axon terminals or pedicles (Supplementary Movie 1).

**Figure 2:**
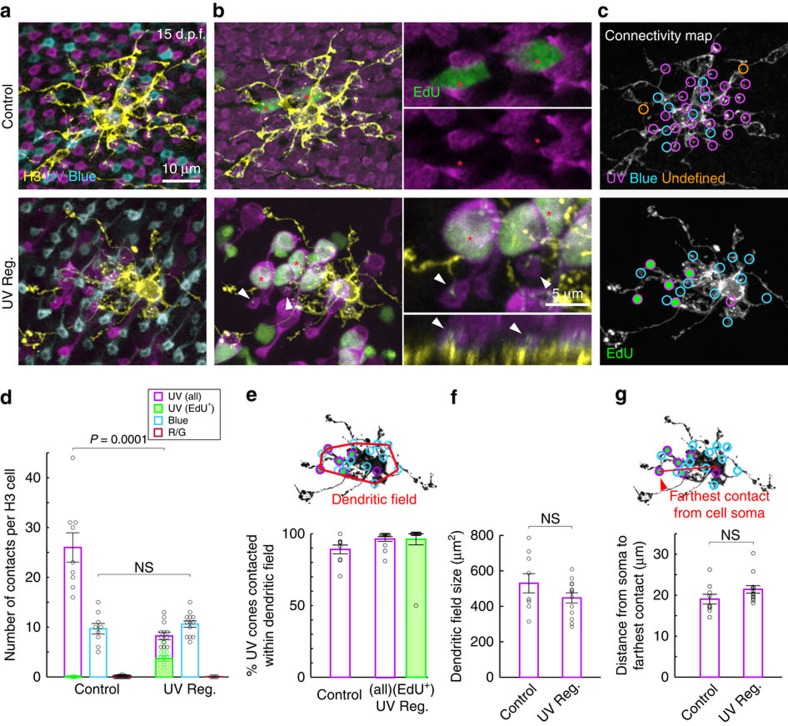
H3 HCs rewire selectively with new ultraviolet cones. (**a**) H3 HCs (yellow) in age-matched control and ultraviolet regenerated (UV Reg.) retinae at 15 d.p.f. visualized by one-cell stage DNA plasmid injection in the background of *Tg(sws1:nfsB-mCherry, sws2:GFP)* retina. Only the pedicles of the cones are shown here. (**b**) EdU (green) does not label ultraviolet cones in control retina (red asterisks), whereas many ultraviolet cones (red asterisks) are EdU positive after a period of regeneration (UV Reg.). The cell bodies and axon pedicles of the ultraviolet cones are shown. In the UV Reg. retina, orthogonal view of the H3 HC reveal dendritic tips inserting into EdU-positive regenerated ultraviolet cones (arrowheads). (**c**) Circles indicate the location of dendritic tips contacting each cone type. Green-filled circles are EdU-positive cones. Dendritic tips not associated with either ultraviolet and blue cones are assigned as undefined. (**d**–**g**). Quantitation of population data. (**e**) The dendritic field is defined as a polygon connecting the outermost dendritic tips. (**g**) Distance from the centre of mass of the cell soma to the farthest dendritic tip is quantified here. For all cells measured, the farthest contact was with an ultraviolet cone. For all plots, circles indicate values from individual cells. Error bars are s.e.m. NS, not significant; (**d**) *P*=0.42, (**f**) *P*=0.35, (**g**) *P*=0.12. *P* values are from Mann–Whitney rank-sum test. UV, ultraviolet.

In regions of ultraviolet-cone regeneration (UV Reg.), many ultraviolet cones were clearly labelled by EdU (Fig. 2a,b). Occasionally, there were EdU-positive cells in the vicinity of H3 HCs in control retina at this age. These EdU-positive cells, however, were not ultraviolet cones and were most likely rod photoreceptors, which are known to be produced throughout larval development^14^. At 15 d.p.f., H3 HCs in control animals form connections with ultraviolet and blue cones, with a biased contact ratio up to 5:1 ultraviolet to blue-cone contacts (Fig. 2a,c,d). Because regeneration did not completely reproduce the wild-type ultraviolet-cone density in Met-treated animals, H3 HCs contacted far fewer cones in the region of regeneration compared with controls (Fig. 2a-d). H3 HCs contacted all ultraviolet cones within their dendritic fields regardless of the number of ultraviolet cones available (EdU positive or negative) (Fig. 2e), and did not extend their dendrites farther than normal to increase connectivity with distant ultraviolet cones (Fig. 2f,g). Moreover, H3 HCs did not compensate for the reduced availability of ultraviolet cones by contacting more blue cones than normal (Fig. 2d). This finding is in stark contrast from previous observations, which showed that H3 HCs increase connectivity with blue cones when very few ultraviolet cones are generated during development^8^. However, it remains possible that when ultraviolet cones are absent immediately after ultraviolet-cone ablation, H3 HCs transiently increase contact with blue cones, but retract these contacts upon ultraviolet-cone regeneration. To investigate this possibility, we performed *in vivo* multiphoton time-lapse imaging to monitor H3 HCs and their contacts with cone photoreceptors in untreated and Met-treated animals at more frequent time intervals.

**Figure 3:**
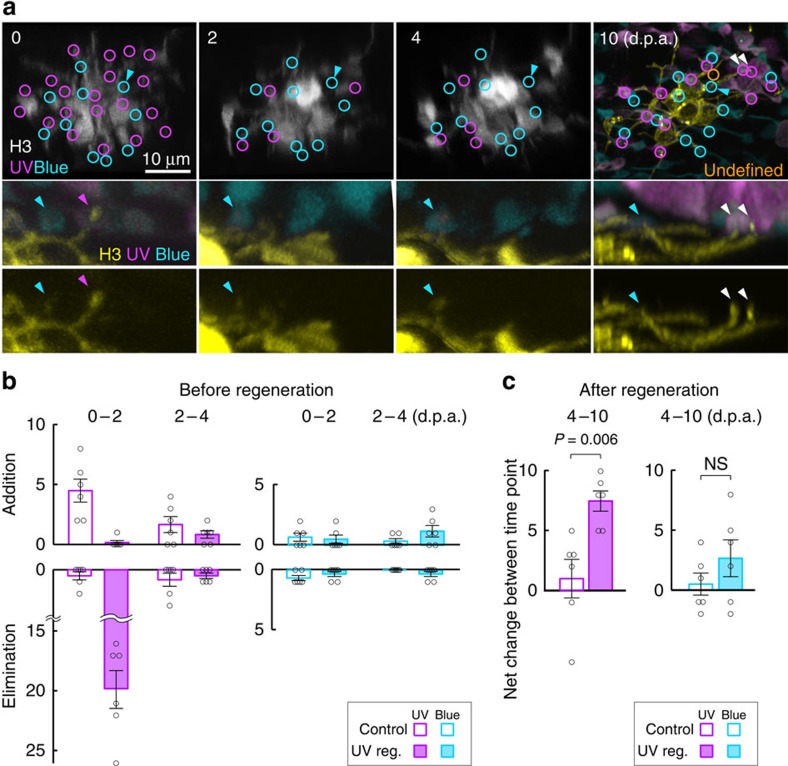
H3 HCs do not elaborate dendrites to contact more blue cones before ultraviolet-cone regeneration. (**a**) Two-photon time-lapse imaging of an H3 HC (grey scale or yellow cell) in the background of *Tg(sws1:nfsB-CFP, sws1:nfsB-mCherry, sws2:mCherry)* before and during ultraviolet-cone regeneration. d.p.a., days post ablation. Cyan arrowheads point to a dendritic tip invaginating a blue-cone axon pedicle that was maintained throughout the time course of imaging. Magenta arrowheads indicate a dendritic tip that retracted after ultraviolet-cone ablation. New dendritic tips elaborated to contact regenerated ultraviolet cones (white arrowheads). Undefined (orange circle); dendritic tip not apposed to blue or ultraviolet cone. (**b**) Addition and elimination of cone contacts across time points before ultraviolet-cone regeneration (*n*=6 cells each for control and regeneration condition (UV Reg.)). Individual cone contacts were followed at 2-day intervals to identify contact appearance or disappearance. (**c**) Net change in cone contact numbers of the same set of cells in **b**, after ultraviolet-cone regeneration. Contacts were visualized in fixed tissue at 10 d.p.a. because pigmentation of the larval retina between 4 and 10 d.p.a. (9–15 d.p.f.) prevented *in vivo* imaging. (**b**–**c**) Circles indicate values from individual H3 HCs. Error bars are s.e.m. NS, not significant (*P*=0.34). *P* values are from Mann–Whitney rank-sum test. UV, ultraviolet.

Daily imaging of H3 HCs at stages before ablation until cone regeneration revealed that following ultraviolet-cone death, most H3 HC dendritic tips retract, whereas some dendrites extend transiently beyond the outer plexiform layer (Supplementary Fig. 2). Upon ultraviolet-cone regeneration, new tips form and contact the regenerated cones. Non-ultraviolet-cone-associated tips that were present at the onset of imaging were relatively stable, and there was no increase in such dendritic tips over time (Supplementary Fig. 2). To directly ascertain whether or not H3 HCs transiently increase connections with blue cones before ultraviolet-cone regeneration, we carried out three-colour, *in vivo* multiphoton imaging to simultaneously visualize H3 HC dendrites (yellow fluorescent protein, YFP), ultraviolet (*Tg(sws1:nfsB-mCherry; sws1:nfsB-CFP*)) and blue cones (*Tg(sws2:mCherry*) (Fig. 3)). Addition and elimination of contacts before ultraviolet-cone regeneration were identified by tracking individual cone contacts with the HC across time points; connections with blue cones did not increase significantly before new ultraviolet cones appeared in Met-treated fish when compared with age-matched controls (Fig. 3b). We confirmed that the HCs we tracked eventually regained connections with regenerated ultraviolet cones, and that they did not increase blue-cone connections overall, by calculating the net change in ultraviolet- and blue-cone connections after the period when ultraviolet cones have regenerated (between 4 and 10 d.p.a.; Fig. 3c). The time-lapse imaging experiments further revealed that when ultraviolet cones survived Met treatment, H3 HCs maintained contact with the surviving cones. These H3 HCs established connections with all ultraviolet cones within their dendritic fields after ultraviolet-cone regeneration (10 d.p.a.), regardless of the number of contacts maintained with surviving ultraviolet cones (Supplementary Fig. 3). This observation suggests that the maintenance of connections with surviving ultraviolet cones does not influence H3 HC synaptogenesis with the population of regenerated ultraviolet cones.

### HCs misconnect if ultraviolet-cone regeneration is delayed

**Figure 4:**
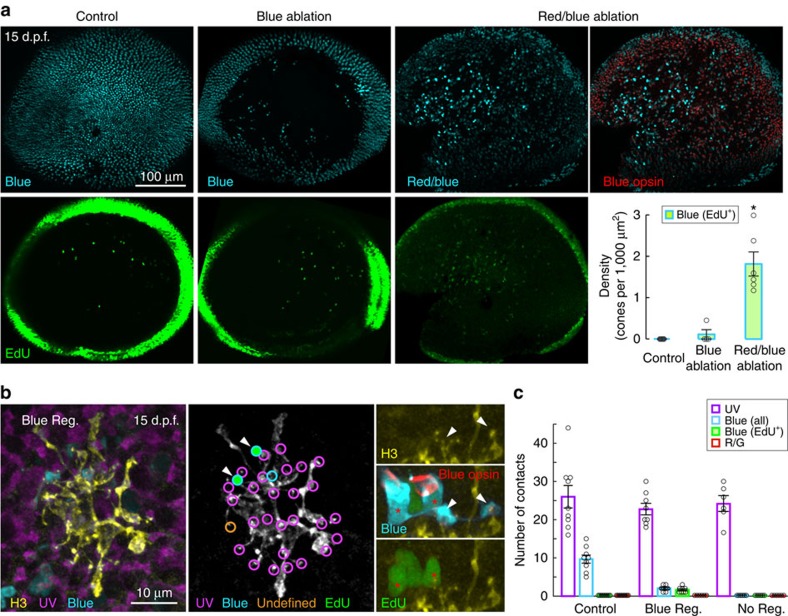
H3 HCs wire with regenerated population of blue cones. (**a**) Distribution of blue cones or red and blue cones in the whole-mount retinae at 15 d.p.f. Blue-cone-only ablation (B ablation) was performed using *Tg(sws2:nfsB-mCherry)*, or blue and red cones were ablated (R/B ablation) together using *Tg(sws2:nfsB-mCherry, thrb:gal4, clmc:GFP; UAS:nfsB-mCherry)*. Blue-opsin immunostaining (red) identified blue cones among red and blue cones that both express mCherry. EdU labelling is shown in green. Density of EdU^+^ blue cones are plotted for all conditions. Circles are values from individual retina. Error bars are s.e.m. *P=0.0022 for pair-wise comparisons with control or R/B ablation. *P* values are from Mann–Whitney rank-sum test. (**b**) An H3 HC in a retina with blue-cone regeneration (Blue Reg.) after red and blue-cone ablation at 5 d.p.f. using *Tg(sws1:GFP, sws2:nfsB-mCherry, thrb:gal4, clmc:GFP; UAS:nfsB-mCherry)* animals. Blue cones were identified by blue-opsin immunostaining (red). Dendritic tips (arrowheads) of the HC invaginated the EdU^+^ blue cones (red asterisks). (**c**) Quantification of ultraviolet and blue-cone contacts in retina in which red and blue cones were ablated. H3 HCs located in retinal regions lacking blue-cone regeneration (No Reg.) are grouped separately from H3 HCs that were located in areas containing regenerated blue cones (Blue Reg.). Red/green (R/G) cones identified by zpr1 immunostaining. Circles are values obtained from individual cells. Error bars are s.e.m. UV, ultraviolet.

**Figure 5:**
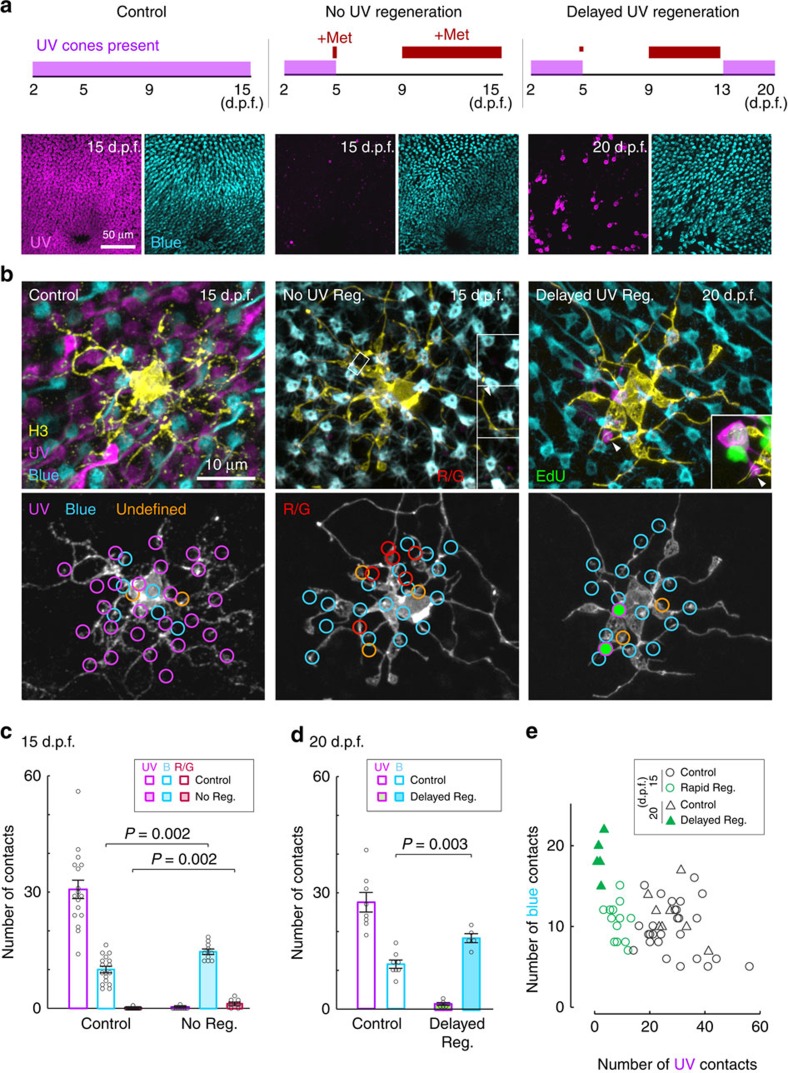
H3 HCs synapse with blue cones and other cone types when ultraviolet cones fail to regenerate. (**a**) Schematic showing period of metronidazole (Met) treatment (red lines) and ultraviolet-cone presence in the retina (magenta bars). Images show distribution of ultraviolet (magenta) and blue (cyan) cones in the whole-mount retina at the end of the treatments. (**b**) Examples of an H3 HC in control retina, in a retina with no ultraviolet regeneration (No UV Reg.) and in a retina with delayed regeneration (Delayed UV Reg.). Inset for the no regeneration example shows sideview of a dendritic tip (arrowhead) in the boxed region contacting a zpr1-positive red or green cone (R/G, shown in red). Inset for the delayed regeneration example shows contact with a new (EdU^+^, green) ultraviolet cone. (**c**,**d**) Measurements across H3 HC populations; circles are measurements from individual cells. Error bars are s.e.m. Mann–Whitney rank-sum test. (**e**) Comparison of the number of ultraviolet- versus blue-cone contacts for individual H3 cells in control and regenerated conditions. UV, ultraviolet. B, blue.

During development, H3 HCs will increase connectivity with blue cones if ultraviolet cones are absent^8^. Thus, it was surprising that ultraviolet-cone ablation after 5 d.p.f. did not induce blue-cone synaptogenesis. Is this because H3 HCs lose their ability to recognize and contact blue cones after their initial circuit is established? We first investigated whether mature H3 HCs can target ‘new' blue cones by ablating this cone type to trigger their regeneration. We found that Met treatment of *Tg(sws2:nfsB-mCherry)* in which blue cones express NTR did not trigger much regeneration (Fig. 4a), and thus we ablated both red and blue cones together using *Tg(sws2:nfsB-mCherry; thrb:gal4, clmc:GFP; UAS:nfsB-mCherry)* quadruple transgenic fish. Concurrent red and blue-cone ablation induced robust regeneration of blue (and red, not shown) cones, without affecting ultraviolet-cone numbers (control: 3.9±0.2 cones per 100 μm^2^, blue- and red-cone ablation: 4.0±0.2 cones per 100 μm^2^, *n*=6 retinas each; Fig. 4a; Supplementary Fig. 4). In these fish, H3 HCs connected with new blue cones present in their vicinity (Fig. 4b). Thus, H3 HCs can form connections with regenerated blue cones after development (>5 d.p.f.). Despite not contacting any, or contacting very few blue cones, H3 HC did not alter their connections with ultraviolet cones (Fig. 4c). Also, there was no compensation for a loss of blue-cone connections by synaptogenesis with red or green cones (Fig. 4c). We next tested the hypothesis that H3 HCs are unable to form additional connections with blue cones that existed before the ablation. To explore this possibility, we prevented repopulation of ultraviolet cones by exposing fish to Met at 5 d.p.f. (1 day) and repeated the treatment between 9 and 15 d.p.f. (Fig. 5a). Met treatment was minimized to avoid bystander death of Müller glia that do not express *nfsb* (data not shown). In the continual absence of ultraviolet cones, H3 HCs increased synaptogenesis with existing blue cones (Fig. 5b,c), implying that mature H3 HCs do not lose the ability to recognize and target this cone type. Indeed, H3 HCs also contacted red or green cones in the absence of ultraviolet-cone regeneration, indicating that the HCs maintain the capacity to contact all cone types during regeneration. However, rapid reconnection with ultraviolet cones prevents HCs from mistargeting non-preferred cone types (Figs 2d and 5b,c).

Can H3 HCs target newborn ultraviolet cones after they have rewired with other cone types? To answer this question, we carried out an ablation paradigm whereby regeneration of ultraviolet cones was delayed and permitted only after H3 HCs had contacted many more blue cones than normal (Fig. 5a). In this scenario (delayed regeneration), we found that H3 HCs contacted new ultraviolet cones within their dendritic field (Fig. 5b,d). Thus, H3 HCs maintain their capacity to target ultraviolet cones even after remodelling their wiring pattern. Furthermore, an increase in blue-cone connectivity is not triggered simply by a reduction of ultraviolet-cone connections compared with controls, because H3 HCs increase connections with blue cones when regeneration is delayed, but not when regeneration occurs rapidly (Fig. 5e).

Together, our observations suggest that there is a transient period during which the postsynaptic HC maintains its synaptic preference after loss of its preferred synaptic partner. However, failure to repopulate the retina rapidly with this partner type triggers synaptogenesis not only with the secondary input type but also with inappropriate partners. Thus, rapid replacement of lost synaptic partners could prevent abnormal rewiring of the surviving retinal cells^15^,^16^,^17^.

## Discussion

It is evident that neurons generated after development can integrate into the mature central nervous system (CNS). Adult-born GABAergic interneurons in the olfactory bulb and granule cells in the dentate gyrus form synaptic connections that are characteristic of cohorts produced during development, indicating that new neurons can wire appropriately within an ‘old' network^18^,^19^,^20^,^21^,^22^. Moreover, cells transplanted into the intact or damaged adult mammalian CNS, including the retina, can form synaptic connections that are appropriate, although at times ectopic connections emerge^23^,^24^,^25^,^26^,^27^. While many of these studies have investigated the connectivity of newborn or transplanted cells, our current study provides insight into how surviving neurons reconstruct their circuitry upon regeneration of their preferred presynaptic partners. The highly localized connections of the HC–photoreceptor circuit enabled us to track morphological changes in the HC and map its connectivity *in toto*, before and after specific photoreceptor partners are ablated and subsequently regenerated.

Studies in the past have shown that neurons remodel their dendrites upon loss of their afferents during development^28^,^29^. Likewise, the H3 HCs retract dendritic tips upon ultraviolet-cone death, although the size of their arbours is unchanged. The lateral confinement of H3 HC dendritic changes during regeneration may not be surprising, because H3 HC dendritic territories are invariant to the number of ultraviolet cones in the field during development^8^. Similarly, mouse HC dendritic arbours maintain their normal size in the ‘conefull' or ‘coneless' mutants^30^,^31^, suggesting that presynaptic contact does not dictate the overall dendritic arbour size of the HCs. We also observed transient dendritic sprouting into the photoreceptor layer as ultraviolet cones were dying, reminiscent of HCs in mouse mutants in which photoreceptors are non-functional or undergo degeneration^32^,^33^,^34^,^35^,^36^. However, unlike in mice where dendrite remodelling can lead to ectopic synaptogenesis^37^,^38^,^39^,^40^, zebrafish H3 HCs rapidly retract their sprouted neurites before elaborating dendritic tips from existing branches to form connections with newborn cells of their preferred partner type. Further, H3 HCs maintain synaptic contact with blue cones during dendritic retraction and extension. Thus, re-establishment of the H3 HC circuit arises from minimal remodelling of the postsynaptic cell, a condition that optimizes reassembly of circuitry. With prolonged loss of ultraviolet cones, H3 HCs remodel their connectivity, forming ectopic synapses with red or green cones. Ectopic connections onto the dendrites of surviving neurons have indeed been observed in other systems in which a major synaptic partner is lost at maturity either due to lesions^41^ or neuronal degeneration^38^,^39^.

**Figure 6:**
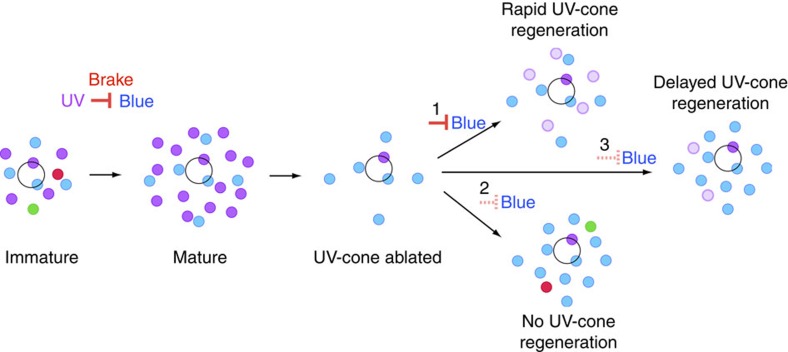
Summary showing consequences of ultraviolet-cone loss and replacement in shaping the connectivity of H3 HCs from development to regeneration. Schematic of H3 HC connectivity patterns during development and regeneration. Colour-filled circles represent cones (purple, ultraviolet cone; cyan, blue cone; green, green cone; and red, red cone) contacted by an individual H3 HC (cell body of HC denoted by open circle). Dark-purple-coloured circles represent original ultraviolet cones; light purple indicate regenerated ultraviolet population. Our previous study suggests that transmission from ultraviolet cones suppresses H3 HC synaptogenesis with blue cones during development^8^. Here we propose that this ‘brake' signal persists after ultraviolet-cone loss for a defined period of time (1), but is lost or not replenished if there is no regeneration of ultraviolet cones (2) or regeneration is delayed (3). UV, ultraviolet.

Our current findings emphasize that there may be a time limit on the ability of the postsynaptic cell to recapture its unique pattern of connections during circuit repair, after which ectopic synapses are formed and circuit remodelling occurs. Our previous finding that ultraviolet-cone transmission restricts synaptogenesis with blue cones during circuit maturation leads us to propose a mechanism that sets the time limit on selective synaptogenesis with ultraviolet cones during circuit regeneration. It is possible that a suppressive signal evoked by ultraviolet-cone transmission during development persists after ultraviolet-cone ablation to maintain the H3 HCs' synaptogenic preference for this cone type. One mechanism by which this can occur is that ultraviolet-cone transmission continually activates a transcriptional pathway that puts a ‘brake' on synaptogenesis with blue cones (Fig. 6). This signal would persist until transcription is downregulated when ultraviolet cones do not reappear. We also found that within 7 d.p.a., H3 HCs did not eliminate excess blue-cone contacts after connecting with some late-arriving ultraviolet cones. This may be because miswiring is irreversible, or perhaps the low numbers of late-born ultraviolet cones were insufficient to generate an elimination signal. Future experiments providing insight into the ‘reversibility' of inappropriate wiring upon increasing the number of regenerated photoreceptors and permitting longer periods of recovery would be immensely helpful in determining whether circuit remodelling after damage can be corrected.

Our observations underscore two other major challenges that need to be overcome to restore CNS circuits to their original wiring patterns. First, recovery of the ultraviolet-cone population was only partial. Second, cell replacement was not specific—other cone types and possibly other neuronal cell types were also generated together with the new ultraviolet cones, potentially altering existing, undamaged circuits. Thus, future cell replacement and circuit repair strategies will need to develop approaches to replace lost synaptic partners completely and, importantly, before remodelling of the surviving circuits. The insights gained here by capitalizing on the intrinsic capacity of zebrafish to regenerate its neuronal populations could in the future extend to other vertebrates, given that recent work has shown that reprogramming of Müller glial cells into progenitors is possible in mice^42^.

## Methods

### Cell-type visualization

Zebrafish were maintained and experiments conducted in accordance with University of Washington Institutional Animal Care and Use Committee guidelines. We used a combination of transgenic lines as listed below to visualize ultraviolet, blue and red cones by FP expression.

*Tg(sws1:GFP)* labels ultraviolet cones^10^ and *nfsB* tagged with FP (*nfsB-FP*) is expressed in ultraviolet cones in *Tg(sws1:nfsB-mCherry)* and *Tg(sws1:nfsB-CFP)* fish. *Tg(sws2:GFP)*^43^ and *Tg(sws2:mCherry)*^44^ label blue cones, and *Tg(sws2:nfsB-mCherry)* transgenic lines express *nfsB-FP* in blue cones. A transgenic line with *nfsB-FP* expression in red cones, *Tg(thrb:gal4; clmc:GFP; UAS:nfsB-mCherry)*, was generated by crossing *Tg(thrb:gal4; clmc:GFP)* with *Tg(UAS:nfsB-mCherry)*^11^; a green fluorescent protein (GFP) heart marker driven by cardiac myosin light chain (*clmc*) promoter^45^ was used to aid the screening of *gal4*-positive fish*. Tg(sws1:nfsB-mCherry)*, *Tg(sws1:nfsB-CFP)*, *Tg(sws2:nfsB-mCherry)* and *Tg(thrb:gal4; clmc:GFP)* transgenic lines were generated by injecting DNA plasmids pSws1:nfsB-mCherry, pSws1:nfsB-CFP, pSws2:nfsB-mCherry or pCG2Thrb:gal4, respectively, into fertilized eggs at the one-cell stage, and progeny were screened by FP expression.

### Plasmid construction

To construct the pUAS:MmTFP1myc plasmid, mTFP1 was exchanged with YFP in the pUAS:MYFP plasmid, and a myc tag was added to the C terminus of MmTFP1. We used the Tol2 kit^45^ to construct the following plasmids. pCx55.5:TrpR plasmid was generated in a Gateway recombination reaction: p5E:Cx55.5, pME:TrpR^46^, p3EpA and pDestTol2CG2. p5E:tUAS, pME:MmTFP1myc, p3E:pA and pDestBleedingHeart were similarly combined for ptUAS:MmTFP1myc plasmid construction. p5E:Cx55.5 was generated by inserting a cx55.5 promoter fragment from the pCx55.5:gal4 plasmid into p5E plasmids^47^. The MmTFP1myc fragment was excised from pUAS:MmTFP1myc and inserted into pME plasmids to generate pME:MmTFP1myc.

We constructed pSws1:nfsB-mCherry, pSws1:nfsB-CFP, pSws2:nfsB-mCherry and pCG2Thrb:gal4 DNA plasmids for transgenic fish genesis. These plasmids were made using the Gateway system, with combinations of p5E:sws1, pME:nfsB-mCherry, p3E:pA and pDestTol2pA for generating pSws1:nfsB-mCherry; p5E:sws1, pME:nfsB-CFP, p3E:pA and pDestTol2pA for producing pSws1:nfsB-CFP; p5E:sws2, pME:nfsB-mCherry, p3E:pA and pDestTol2pA for pSws2:nfsB-mCherry; p5E:thrb^48^, pME:gal4, p3E:thrb and pDestTol2CG2 for pCG2Thrb:gal4. pME:nfsB-mCherry was generated by inserting a nfsB-mCherry fragment into pME plasmids. mCherry was exchanged with cyan fluorescent protein (CFP) to produce pME:nfsB-CFP plasmids.

### Cone ablation

Zebrafish were immersed into system water containing Met to ablate nfsB-expressing cones. For acute ultraviolet-cone ablation, 5 d.p.f. fish were immersed in a final concentration of 10 mM Met for 2 h. For acute ablation of red and blue cones, 5 dpf fish were immersed in 5 mM Met for 1 hour. In prolonged ultraviolet-cone ablation experiments, fish were immersed in 5 mM Met from 9 to 13 d.p.f. or 9 to 15 d.p.f., and half of the volume of Met solution was exchanged every other day. Following Met treatment, zebrafish were transferred into system water without Met. Zebrafish were fed regularly during prolonged Met treatment.

### BrdU and EdU labelling

To label dividing cells in live zebrafish, BrdU or F-ara-EdU^49^ was added to the system water to a final concentration of 1 or 0.5 mM, respectively. Incubation was timed according to the experimental paradigm. At the end of the treatment period, zebrafish were transferred into system water without BrdU or EdU and either raised normally or fixed immediately.

Zebrafish were killed by icing, and then fixed in a solution of 4% paraformaldehyde and 3% sucrose in 0.1 M PBS, pH 7.4, for 30 min at room temperature. Samples were washed three times for 5 min in PBS. Eyes were dissected in PBS, and the cornea, lens and pigmented epithelium were removed. For BrdU immunostaining, retinal tissue was permeabilized in PBS buffer containing 1% Triton-X100, 1% Tween-20 and 1% dimethylsulphoxide (PBTTD) for >30 min, and treated with DNase I in PBTTD for 30 min at 37 °C to facilitate antibody access. After three PBTTD washes, the tissue was immersed in blocking solution containing 5% normal donkey serum in PBTTD, for 1–12 h at 4 °C, and incubated with anti-BrdU antibody (mouse, 1:50, BD Cat. 347520) in blocking solution for 1–3 days at 4 °C. The tissue was washed three times in PBTTD, followed by secondary antibody incubation, anti-mouse IgG DyLight649 (goat, 1:1000, Jackson ImmunoResearch) in blocking solution, for 2–5 h at room temperature. Subsequently, the tissue was washed three times with 0.5% TritonX-100 in PBS, hemisected by a scalpel blade (No.11, Feather), mounted in agarose and covered with Vectashield mounting solution under a coverslip.

For EdU labelling, whole retinae were permeabilized by 0.5% TritonX-100 in PBS for >30 min at 4 °C, followed by three PBS washes for 5 min. Click-it reactions were carried out in PBS solution with sodium ascorbate (Sigma, Cat. A7631; 20 mM), copper(II) sulfate (Sigma, Cat. 451657; 2 mM) and Cy5-azide (Lumiprobe, Cat. A3030; 10 μM) for 30 min at room temperature. After three PBS washes, samples were processed for immunohistochemistry.

### Immunohistochemistry

Zebrafish were fixed for 30 min in 4% paraformaldehyde and 3% sucrose in 0.1 M PBS at room temperature. Retinae were dissected as described in the section BrdU and EdU labelling. Tissues were blocked in PBS containing 5% normal donkey serum and 0.5% TritonX-100 for >30 min at 4 °C, and incubated in primary antibody in blocking solution for 2–5 days at 4 °C. After washing three times with 0.5% TritonX-100 PBS, retinae were incubated in secondary antibody for 2 h at room temperature. After three washes in PBS with 0.5% Tritonx-100, retinae were mounted in agarose and coverslipped in Vectashield (Vector Labs). Primary antibodies were as follows: zpr1 (mouse, 1:100, ZIRC), anti-GFP (chicken, 1:500, Abcam ab13970), anti-blue-opsin (rabbit, 1:2,000, kindly provided by Jeremy Nathans) and anti-myc (mouse, 1:100, DSHB 9E10). Secondary antibodies were as follows: anti-mouse IgG DyLight649 (goat, 1:1,000, Jackson ImmunoResearch), anti-mouse IgG DyLight405 (donkey, 1:1,000, Jackson ImmunoResearch), anti-rabbit IgG DyLight405 (donley, 1:1,000, Jackson ImmunoResearch) and anti-chicken IgY DyLight488 (goat, 1:1,000, Jackson ImmunoResearch).

### Two-photon imaging

Fish embryos were treated with 0.1 mM 1-phenyl 2-thiourea (PTU) typically from 24 h post fertilization (h.p.f.) until 4 d.p.f. to prevent pigmentation and allow screening for FP expression. For time-lapse imaging, fish were treated with PTU from 10 h.p.f. until the final time point of *in vivo* imaging. Larval zebrafish were mounted in 1% low-melting-point agar (Sigma, Type VII agarose) in system water containing 0.1 mM PTU and 25 mg l^−1^ MS-222. Images were acquired using a custom-built two-photon microscope equipped with a Ti-Sapphire tunable infrared laser (Spectra-Physics). Two-colour imaging of YFP and mCherry fluorescence was conducted at 940-nm laser wavelength. Three-colour imaging was achieved by scanning the area of interest twice, first at 940 nm to image YFP and mCherry, and second at 900 nm to image CFP and YFP together. The two image stacks were aligned using AMIRA software (FEI). Images were taken with an XY resolution of 0.07 μm per pixel and 0.3 μm Z steps using a 1.1 numerical aperture (NA) × 60 water-immersion objective lens (Olympus). After image acquisition, fish were released from agar and returned to a solution of 0.1 mM PTU in system water.

### Confocal image acquisition

Image stacks were acquired on a confocal microscope (Olympus FV1000) using a 1.35 NA × 60 oil-immersion or 0.85 NA × 20 oil-immersion lens. High-magnification images were typically obtained at a resolution of 0.07 μm per pixel XY and 0.25 μm Z step, and low-magnification images were acquired at a resolution of 0.42 μm per pixel XY and 0.5 μm Z step.

### Image analysis

All image stacks were median filtered in Fiji^50^. Maximum intensity projections of the regions of interest were visualized in Fiji (NIH) or Amira (FEI), respectively. Three-dimensional-reconstructed images were oriented and digitally sliced using the oblique slice function in Amira. Image brightness, contrast and hue were further adjusted in Photoshop (Adobe). Cone densities were analysed by counting the number of labelled cones within an area in central retina, of a size corresponding to the retina generated by 5 d.p.f. Cones were counted by a custom, semi-automated method in Matlab.

### Statistical analysis

We used the Wilcoxon–Mann–Whitney rank-sum test for all comparisons.

## Additional information

**How to cite this article:** Yoshimatsu, T. *et al.* Presynaptic partner selection during retinal circuit reassembly varies with timing of neuronal regeneration in vivo. *Nat. Commun.* 7:10590 doi: 10.1038/ncomms10590 (2016).

## Supplementary Material

Supplementary Information Supplementary Figures 1-4 and Supplementary Reference

Supplementary Movie 1 In vivo multiphoton reconstruction of a YFP labeled H3 HC at 5 dpf in the background of a transgenic animal in which UV cones were labeled by expression of mCherry under the opn1sw1 promoter. Dendritic tip invaginations into the axon pedicles of the UV cones are more readily viewed upon digital slicing through the image stack.
