# Supplementary material for: Presynaptic partner selection during retinal circuit reassembly varies with timing of neuronal regeneration in vivo
Source: Nat Commun. 2016 Feb 3;7:10590. doi: 10.1038/ncomms10590 (PMC4742908; doi:10.1038/ncomms10590)
Supplement: Supplementary Information — Supplementary Figures 1-4 and Supplementary Reference [file ncomms10590-s1.pdf]

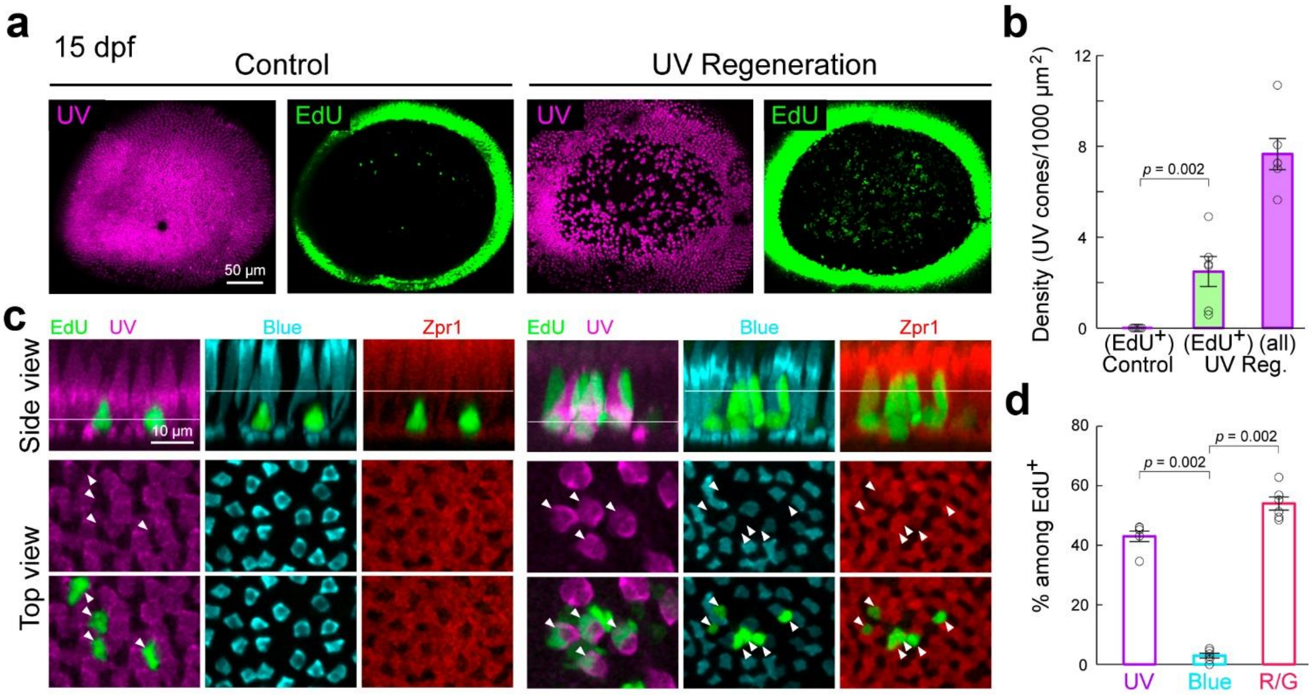

Supplementary Figure 1. UV cone ablation induces regeneration of specific cone types.

(a) Wholemount view of UV cone (magenta) distributions at 15 dpf in a control retina (no ablation) and in a retina after UV cone ablation at 5 dpf *Tg(sws1:nfsB-mCherry)*. EdU (green) was applied to the animals from 1-4 dpa. Dense ring of EdU$^+$ cells mark the ciliary marginal zone where cell genesis persists into adulthood[1].

(b) Densities of EdU$^+$ UV cones at 15 dpf plotted for control retina and retinae with UV cone regeneration (UV Reg.). The density of all UV cones (EdU$^+$ or EdU$^-$) present in the UV reg. retina are also plotted. Each circle represents measurements from one retina.

(c) (Side view) Orthogonal views of confocal image stacks from control and UV cone ablated retinae showing colabeling for various cone types and EdU (green). zpr1 positive cells (red) are red or green cones. The levels at which UV, blue and red/green cone nuclei stratify are indicated by their respective lines. (Top view) *En face* view of the cones and EdU labeled nuclei at the levels of the lines marked in the sideviews. Arrowheads point to EdU$^+$ nuclei.

(d) Percentage of EdU$^+$ cones that were UV, blue or red/green comprising the regenerated population.

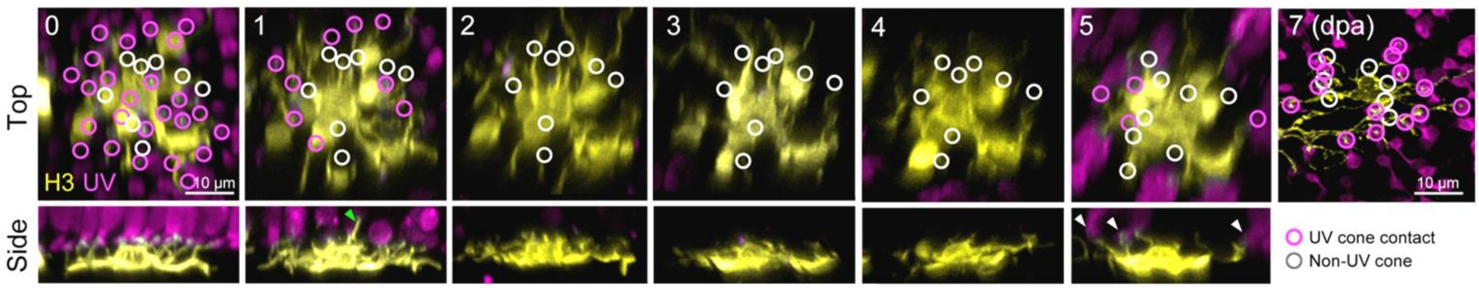

Supplementary Figure 2. H3 HCs retract dendritic tips as UV cones die, and rewire with the regenerated population of UV cones.

Two-photon time-lapse imaging of a H3 HC in the background of *Tg(sws1:nfsB-mCherry)* beginning at 5 dfp and subsequently after cone ablation and regeneration. Circles indicate the locations of dendritic tips contacting UV cones (magenta) or dendritic tips that were not associated with UV cones (white, non-UV cones). Green arrowhead (1 dpa) points to a dendrite sprouting into the photoreceptor layer. White arrowheads (5 dpa) indicating new dendritic contacts with newly generated UV cones. dpa, days postablation.

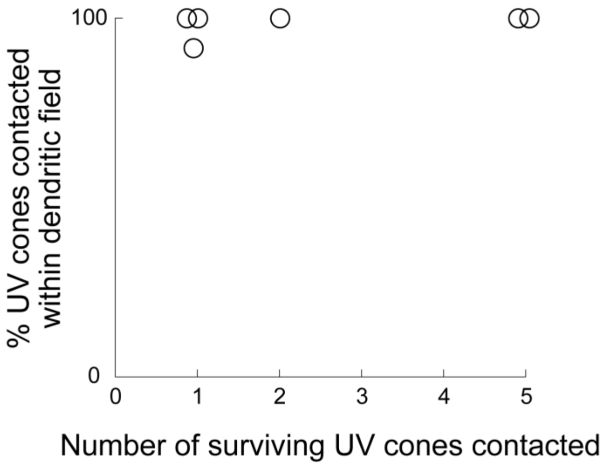

Supplementary Figure 3. Surviving UV cones do not influence synaptogenesis of H3 HCs with regenerated UV cones.

The percentage of UV cones available within the H3 HC dendritic territory that were contacted by the cell is plotted as a function of the number of connections with surviving UV cones. Circles indicate measurements from individual cells.

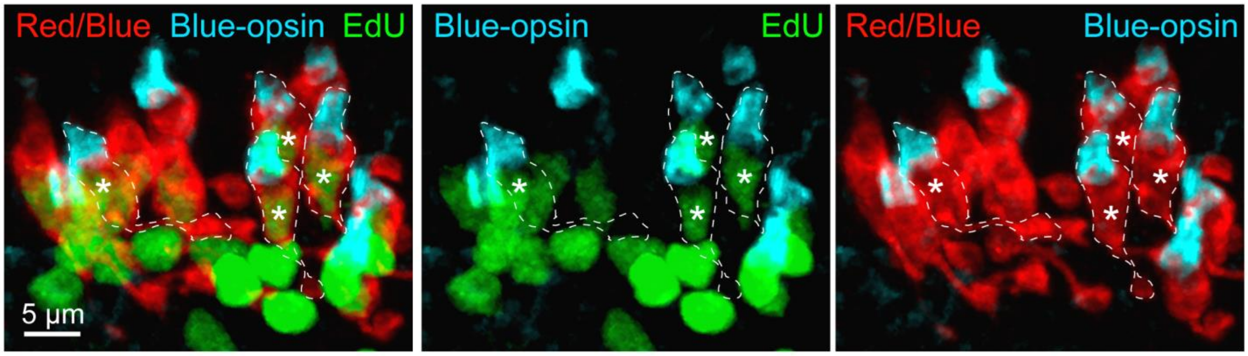

Supplementary Figure 4. Identification of regenerated blue cones.

Blue opsin immunostaining and EdU labeling of *Tg(sws2:nfsB-mCherry; thrb:gal4; clmc:GFP; UAS:nfsB-mCherry)* retina. Blue opsin signal identifies blue cones among mCherry positive red and blue cones. EdU positive blue cones are outlined by dashed profiles. Asterisks mark EdU positive nuclei.

Supplementary Reference

1. Johns, P. R. Growth of the adult goldfish eye. III. Source of the new retinal cells. *J. Comp. Neurol.* **176,** 343–357 (1977).
